# Establishment of a successful robotic pediatric general surgery practice

**DOI:** 10.1007/s11701-023-01667-y

**Published:** 2023-07-15

**Authors:** Steven L. Raymond, Fransua Sharafeddin, Marla A. Sacks, Daniel Srikureja, Nephtali Gomez, Donald Moores, Andrei Radulescu, Faraz A. Khan, Edward P. Tagge

**Affiliations:** 1 Division of Pediatric Surgery, Department of Surgery, Loma Linda University School of Medicine, 11175 Campus St, Suite, Loma Linda, CA 21111 USA; 2 Department of Surgery, Memorial Hospital of South Bend, South Bend, Indiana USA

**Keywords:** Robotic surgery, Pediatric surgery, General surgery, Minimally invasive, Robotic-assisted surgery

## Abstract

Robotic-assisted surgery (RAS) has a variety of theoretical advantages, including tremor filtration, optimal visualization, and improvement of surgeon ergonomics. Though it has achieved wide application in pediatric urology, the majority of pediatric general surgeons do not employ RAS. This study reports our institution’s experience with RAS on a pediatric general surgery team. Following IRB approval, a retrospective review of all pediatric patients at our academic children’s hospital who underwent RAS between 2017 and 2022 for pediatric general surgical conditions was performed. Patient demographics, operation performed, operating time, complications, and recovery were evaluated. A total of 159 children underwent RAS, increasing from 10 patients in 2017 to 59 patients in 2022. The median age and weight were 15.3 years and 76.4 kg, and 121 (76.1%) were female. The application of RAS was successful in all cases. There were no intraoperative complications and no conversions to an open approach. Eleven patients (6.9%) had unplanned presentations to the emergency department within 30 days. Five of these patients (3.1%) required admission to the hospital. This study demonstrates that the application of RAS in an academic pediatric general surgery practice is feasible and safe. The application of RAS to pediatric general surgery should continue to increase as operative teams increase their experience and comfort.

*Level of evidence* Level IV.

## Introduction

The use of robotic-assisted surgery (RAS) was first reported in 1988 for image-guided stereotactic neurosurgery [1]. Since this initial report of the use of RAS, the application has been described in various fields of surgery including urology, gynecology, cardiothoracic, gastrointestinal, hepato-pancreato-biliary, orthopedic, and otolaryngology. In 2001, Meininger et al. published the first reported pediatric robotic case in which the team performed a Nissen fundoplication in a 10-year-old female [2].

Implementation of robotic-assisted surgery approaches a new paradigm in treatment methodology of different pediatric conditions. RAS provides broadened possibilities and sets a positive tendency for better outcomes. Technical advancements allow the utilization of robots during surgical interventions with several advantages, including three-dimensional visualization, tremor filtration, and improvement of surgeon ergonomics [3]. Optimal visualization is a critical factor during surgical procedures, allowing more favorable exposure with the identification of vital structures. Assessment of the accuracy of a robotic surgical arm compared to a human surgical assistant during laparoscopic surgeries have demonstrated that robotic devices are capable of better manipulation and more accurate control of the video endoscope, which provides a greater advantage in visualization with potentially better outcomes [4]. Another important advantage of RAS is that implementation of robots allows tremor filtration. High-frequency motions caused by tremor represent a prominent concern for surgeons, specifically when operating in the pediatric population with a substantially limited surgical field, creating a lack of precision. Implementation of robotics in this case allows one to filter these high-frequency motions caused by tremor, via computer motion scaling, which represents a down-scaling of larger movements of a surgeon to much smaller movements of the surgical instrument in the operation field [5]. Likewise, previous case series have suggested that RAS is superior to laparoscopic surgery in the performance of the most challenging operative step [6]. Potential risks and disadvantages specific to the use of RAS include equipment and computer system failure, lack of haptic feedback, and size of robotic surgical instruments.

Although it has been widely implemented and gained great popularity in pediatric urology, robotic-assisted surgeries still have not achieved expansive application in pediatric general surgery [7–13]. In this study, we report our institution’s experience with robotic-assisted surgeries on a pediatric general surgery team. We describe our first 159 consecutive RAS cases over a six-year period.

## Methods

### Setting

Loma Linda University Children’s Hospital is a 364-bed, tertiary care medical center that provides care to more than 1.2 million children in the region and trains pediatric surgery fellows and general surgery residents. The division of pediatric surgery consists of five full-time, board-certified pediatric surgeons and performs over 1500 operations per year. With the assistance of Intuitive Surgical training program and our adult robotic-assisted surgery colleagues, all five pediatric surgeons were successfully credentialed for RAS. The Food and Drug Administration approved Intuitive Surgical da Vinci Xi Surgical System (Intuitive Surgical, Sunnyvale, CA, USA) was applied at the discretion of the pediatric surgeon. The da Vinci Xi Surgical System consists of a surgeon console, patient cart, and vision cart. The patient cart is positioned at the bedside and holds the endoscope and up to three additional robotic instruments. The surgeon’s hand movements at the surgeon console are translated in real-time to the patient cart and associated robotic instruments. The vision cart facilitates communications between all components and supports the 3D high-definition vision system.

### Study design

Institutional review board approval (#5,220,196) was granted by Loma Linda University for this retrospective clinical cohort study, with waiver of informed consent. The Loma Linda University Children’s Hospital Pediatric Surgery database was queried for patients who underwent robotic-assisted surgery for pediatric general surgical conditions from 2017 to 2022. Inclusion criteria included children of the ages 0 to 18 years who underwent robotic-assisted surgery for pediatric general surgical conditions. Exclusion criteria included urologic operations. Patient data was extracted from the electronic medical records including demographic information, operation performed, total operating time, robot console time, complications, and recovery. Total operating time was defined as the time from initial incision to procedure end. This time is inclusive of access, docking, console time, undocking, and closure.

### Data analysis

Data were collected using a standardized form and analyses were performed using Microsoft Excel™ Version 15.38. Data are presented as frequency and percentage for categorical variables and as median and range for continuous variables.

## Results

**Fig. 1:**
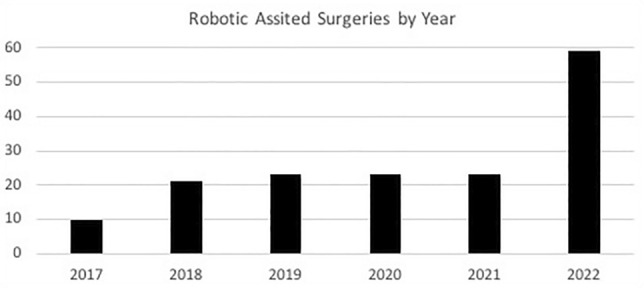
The number of pediatric robotic-assisted surgeries performed each year increased through the six-year study period

A total of 159 children underwent robotic-assisted surgery between 2017 and 2022. The utilization of RAS increased from 10 patients in 2017 to 59 patients in 2022 as shown in Fig. 1. Patient age ranged from 1.6 to 18.2 years with a median of 15.3 years (Table 1). Weight ranged from 10.6 to 158.8 kg with a median weight of 74.8 kg. Overall, 101 patients (63.5%) identified as Hispanic or Latino, and 53 patients (33.3%) identified as Non-Hispanic White or Caucasian. Ethnicities of the remaining patients are listed in Table 1. The majority of patients were female (*n* = 121, 76.1%). Most patients had no previous abdominal surgeries (*n* = 145, 91.2%). The most common comorbidity was obesity (*n* = 86, 54.1%). The majority of patients were classified as American Society of Anesthesiologists (ASA) physical status classification II (*n* = 102, 64.2%).

**Table 1:** Patient Demographics

Characteristic	Overall (*n* = 159)
Age in years, median (range)	15.3 (1.6–18.2)
Weight in kilogram, median (range)	74.8 (10.6–158.8)
Body mass index, median (range)	28.3 (14.1–68.5)
Female, *n* (%)	121 (76.1)
Race, *n* (%)	
Black or African American	3 (1.9)
Hispanic or Latino	101 (63.5)
White, non-hispanic	53 (33.3)
Unknown	2 (1.3)
Weight category, *n* (%)	
Healthy weight	52 (32.7)
Overweight	21 (13.2)
Obesity	40 (25.2)
Severe obesity	46 (28.9)
ASA physical status, *n* (%)	
Class I	40 (25.2)
Class II	102 (64.2)
Class III	17 (10.7)
Class IIV	0 (0)
Previous abdominal surgery, *n* (%)	14 (8.8)

The most frequent RAS was cholecystectomy (*n* = 126, 79.3%) (Table 2). Ten ovarian cystectomies (6.3%), five complex foregut procedures (3.1%), four adrenalectomies (2.5%), four splenectomies (2.5%), and four urachal excisions (2.5%) were performed. The remaining six RAS included distal pancreatectomy with splenectomy, congenital diaphragmatic hernia repair, median arcuate ligament release, excision of pelvic tumor implants, recurrent inguinal hernia repair, and diagnostic laparoscopy (planned colectomy for inflammatory bowel disease aborted due to advanced disease). Complex foregut procedures included two Heller myotomies with Dor fundoplication, one Nissen fundoplication, one paraesophageal hernia repair without fundoplication, and one subtotal gastrectomy for a congenital vascular malformation.

**Table 2:** Robotic Assisted Surgery by Category

Operation	Overall (*n* = 159)
Cholecystectomy, *n* (%)	126 (79.3)
Ovarian cystectomy, *n* (%)	10 (6.3)
Complex foregut, *n* (%)	5 (3.1)
Adrenalectomy, *n* (%)	4 (2.5)
Splenectomy, *n* (%)	4 (2.5)
Urachal excision, *n* (%)	4 (2.5)
Miscellaneous, *n* (%)	6 (3.8)

Among the 126 patients undergoing robotic-assisted cholecystectomy, the median age was 15.6 years (range 10.3–18.2) with a median weight of 79.9 kg (range 42.9–158.8). Eighty patients (63.5%) were Hispanic or Latino. Obesity affected 79 patients (62.7%) undergoing cholecystectomy. Indications for cholecystectomy were symptomatic cholelithiasis in 68 children (54%), gallstone pancreatitis in 25 children (19.8%), calculous cholecystitis in 15 children (11.9%), choledocholithiasis in 14 children (11.1%), and biliary dyskinesia in four children (3.2%). The mean operative time for robotic-assisted cholecystectomies was 110 min. The mean operating time for the first 63 robotic-assisted cholecystectomies was 132 min and 88 min for the most recent 63 robotic-assisted cholecystectomies. The 114th performed robotic-assisted cholecystectomy was the shortest at 40 min. Console times were available for the most recent 51 cases with a median console time of 37 min (range 11–124). Two patients (1.6%) required subtotal cholecystectomies given difficult intraoperative conditions. There were no ductal injuries or bile leaks. Among cholecystectomy patients, 52 (41.3%) were discharged on the day of surgery and 58 (46.0%) were discharged on postoperative day one.

The application of RAS was successful in all cases. There were no equipment or computer system failures, intraoperative complications, conversions to an open approach, or reoperations (Table 3). Among all operations, 62 patients (40.0%) were discharged on the same day as the operation, and 68 patients (42.8%) were discharged on postoperative day one. Within 30 days of the operation, 11 patients (6.9%) had unplanned presentations to the emergency department. Four patients presented with abdominal pain and constipation. Three patients had abdominal pain, direct hyperbilirubinemia, and concern for retained common bile duct stone. The remaining four patients presented with diarrhea, pain and emesis, fever of unknown origin, and incisional pain, respectively. Five total patients (3.1%) required readmission to the hospital within 30 days of the operation. This included the three patients who presented with concerns about retained common bile duct stones: only one required endoscopic retrograde cholangiopancreatography for stone extraction. The other two patients had imaging that failed to disclose retained common duct stones. There were no 30-day mortalities.

**Table 3:** Clinical Outcomes

Outcomes	Overall (*n* = 159)
Equipment failure, *n* (%)	0 (0)
Intraoperative complications, *n* (%)	0 (0)
Conversion to open, *n* (%)	0 (0)
Reoperation, *n* (%)	0 (0)
Unplanned emergency department, *n* (%)	11 (6.9)
Unplanned readmission, *n* (%)	5 (3.1)
Mortality, *n* (%)	0 (0)

## Discussion

Application of RAS in an academic pediatric general surgery practice is feasible and safe. We report 159 consecutive robotic-assisted surgeries performed at a single academic children’s hospital over a six-year period. Five pediatric surgeons with extensive laparoscopic experience but minimal robotic experience were fully trained and credentialed during this period. There were no equipment failures, intraoperative complications, or conversions to open.

Robotic surgery has gained widespread application within the adult population, but the application of robotic surgery for children has occurred much more slowly. Until recently, the majority of the increased robotic application has resided in the pediatric urology, with a case volume increasing an average of 236.6% per year [14]. As a result, the most common robotic-assisted surgeries initially in children were pyeloplasty and ureteral implantation, followed by fundoplication and cholecystectomy.

On first look, the advantages of robotic surgery include many of the same benefits as other minimally invasive techniques, including minimizing operative trauma, decreasing postoperative pain, and potentially reducing hospital stays. The robotic system additionally includes the surgeon console which is separate from the operating table. These facilitate the application for telesurgery, which explains the Department of Defense’s involvement in the development of the first fully functional surgical robot [15]. For the military, there was a need to provide expert surgical care immediately after major trauma. Rather than the traditional “Golden Hour” where the focus was hemorrhage control and prompt evacuation, the military intended to change the paradigm to the “Golden Minute” by bringing the operating room to the casualty for damage control surgery. In addition, dual surgeon consoles facilitate learning by providing immediate surgical supervision by an experienced surgeon.

Robotic-assisted surgery has several technical advantages which may be additive to pediatric applications. Robotic systems are equipped with motion scaling, which reduces the scale of the surgeon’s movements 5:1, supporting detailed movements in small spaces. In addition, the highly magnified three-dimensional images provide a degree of visualization that cannot be achieved by either open or laparoscopic techniques; robotic systems are capable of magnifying images 10–15 times. Potentially more important, the robotic technology provides tremor filtration and operator-controlled views, markedly improving visualization, and operative field dexterity.

As our robotic experience has increased, utilization of RAS has steadily gained popularity among the surgeons. Thirty-seven percent of our robotic cases were performed within the last 12 months of the study. This reflects the full credentialing of five pediatric surgeons, training of a large number of nurses and support staff, minimal morbidity for the initial robotic cases, and apparent superiority of robotic technology for complex pathology.

The increased usage of RAS was spearheaded by the increasing burden of symptomatic biliary tract disease in adolescents. The literature has noted the prevalence of cholelithiasis has risen and ranges from 1.9 to 4% in children [16], and simultaneously the number of performed cholecystectomies has risen by 213% over a 9-year period [17]. This increase may be caused by the worrisome problem of childhood obesity. According to the Centers for Disease Control and Prevention, the prevalence of childhood obesity for 2017–2022 was 19.7% affecting about 14.7 million children and adolescents [18]. Childhood obesity is highest among adolescents (22.2%) and Hispanics (26.2%).

In our series, robotic technology was most frequently applied to adolescents with symptomatic cholelithiasis. We performed robotic-assisted cholecystectomy in 126 patients, including 53 patients in 2022. Indications for cholecystectomy were symptomatic cholelithiasis (54.0%), gallstone pancreatitis (19.8%), choledocholithiasis (11.1%), calculous cholecystitis (11.9%), and biliary dyskinesia (3.2%). Eighty patients (63.5%) who underwent robotic-assisted cholecystectomy were Hispanic or Latino. The median age was 15.6 years and the median weight of 79.9 kg, with obesity affecting 62.7%. In this challenging patient population, there were no conversions to either laparoscopic or open techniques. Two patients underwent unplanned robotic subtotal cholecystectomies for severe acute disease. There were no ductal injuries or bile leaks. Increasing experience has led to a marked decrease in operative times. The mean operating time for the first 66 cholecystectomies was 132 min and for the most recent 63 robotic-assisted cholecystectomies was 88 min, which represents a 33% decrease. Among cholecystectomy patients, the overwhelming majority (87.3%) were discharged within one postoperative day.

Comfort and increased facility with robotic technology have allowed us to venture into more challenging pediatric disease processes, including achalasia and solid tumor extraction. Some authors feel that robotic Heller myotomy is superior to open or laparoscopic, because of a superior three-dimensional image and the use of articulating instruments which enable parallel entry to the submucosal field [19]. With the advantage of enhanced operative field visualization and instrument control, robotic adrenalectomy also appears to be feasible and safe [20].

Robotic general surgery offers multiple potential benefits over open and laparoscopic surgery including enhanced precision, improved visualization, and better surgeon ergonomics. However, the impression that robotic surgery is more expensive compared to ‘traditional’ surgical approaches has hindered its application. The most prohibitive limitation to the widespread utilization of robotics is the initial cost of the robotic surgical system (surgeon console, patient cart, and vision cart), which costs $0.6–2.5 million [21]. Pediatric surgeons, with an anticipated lower case volume, may overcome initial costs by sharing a robotic surgical system with their adult surgeon counterparts [22]. However, this may severely limit the block time available for each individual surgeon, and thus prolong the necessary learning curve and thus impact the potential contribution margin. Fortunately, as in many other hospital systems, our children’s hospital had a robotic platform provided by charitable benefactors, thus markedly decreasing the overall cost and encouraging its application.

The remaining costs include fixed costs (equipment depreciation, annual maintenance contract) as well as variable costs (staff costs, robotic supplies, non-robotic supplies). An annual maintenance contract can cost $100,000–170,000 per year [21]. A review of 14 hospitals in the United States and roughly 6000 robotic cases assessed the cost of ownership, which includes initial equipment acquisition, variable supply and case costs, and fixed maintenance costs [23]. Hospitals with an efficient robotic program had an average fixed cost of $984 per case. Weighted average variable costs per case differed based on the specific surgery performed, with an average of $3325 for robotic cholecystectomies. The main cost drivers for variable costs for all cases were non-robotic supplies (50%), staff costs (29%), and robotic supplies (22%). For robotic cholecystectomies, they noted that total operating time averaged 84 min, mean robotic supplies cost $1153, and mean non-robotic supplies cost $763. In our study herein, the average operative time for robotic cholecystectomies was 110 min, and our average total supply costs (non-robotic and robotic supplies) for robotic cholecystectomies was $916 (range $711–1528). Detailed cost data beyond total case supply costs do not exist for pediatric surgery services like ours.

Gaining access to cost data can have a direct and positive effect on a robotic program’s financial success. However, a recent systematic review concluded that the methodological quality of studies evaluating the costs of robotic surgery was low and insufficient to inform action by hospitals [24]. Managerial accounting methods vary significantly between hospitals, and within any given hospital, accounting methods are not standardized for robotic and non-robotic cases—thus making comparisons difficult, if not impossible.

As noted by Denning et al., cost analysis “should be considered as value equating to quality, as defined by positive outcomes/cost” [25]. Equipment costs remain high, but charges related to operative time are dropping, as evidenced by our 33% decrease in robotic cholecystectomy times. Proponents of robotic surgery would argue that it can potentially lead to long-term cost savings. Additionally, as robotic technology advances and competition increases, it is expected that equipment acquisition costs will decrease over time. Other advantages are harder to factor into the financial equation. For instance, robotic surgery is ergonomically superior to both open and laparoscopic surgery [26], which is beneficial to the health of surgeons and may decrease ergonomic injuries. In addition, a robotic surgery curriculum has been demonstrated to enhance minimally invasive general surgery training [27], and a well-organized multidisciplinary robotic program can positively impact an organization’s marketing plan and financial standing [28]. In conclusion, institutions must weigh the initial costs of starting a robotic program with a potential increased surgical complexity, improved surgeon ergonomics, institutional marketing advantages, and accelerated trainee education.

Overall, with increased utilization, robotic operating times have significantly decreased. We are planning a matched comparison of laparoscopic and robotic-assisted cholecystectomies performed during the same period in this same patient population. The majority of patients undergoing RAS were able to be discharged within one postoperative day. As evidenced by less than 7% unplanned emergency department presentations and a 3% readmission rate, pediatric patients can safely undergo RAS and be allowed to complete their recovery at home.

There are limitations of this study that bear discussion. This retrospective analysis is limited in scope by the information documented in the electronic medical record. Furthermore, the study did not investigate patient preference and satisfaction which may impact the utilization of RAS. Surgeon preference for the utilization of robotics may also play a role and introduce selection bias in the study. Patients that had unplanned presentations to other facilities within 30 days of their operation would not have been captured based on this study design. Despite the inherent limitations, this relatively large series of an institution’s consecutive use of robotic-assisted surgery over a six-year period adds to the growing body of literature involving use of robotics in pediatric general surgery.

The application of RAS to pediatric general surgery should continue to increase as operative teams increase their experience and comfort. Future studies should focus on comparing clinical outcomes of general pediatric operations performed by laparoscopic and robotic-assisted surgical approaches.

## Data Availability

All the data obtained and analyzed in this study was conducted by the authors.
